# P-1723. Comparison of Antibiotic Prescribing for Urinary Tract Infections (UTI), Sinusitis, Group A Streptococcal Pharyngitis, and Pyelonephritis by Prescriber Type

**DOI:** 10.1093/ofid/ofae631.1887

**Published:** 2025-01-29

**Authors:** Brianna Vanhouten, Owen Eckardt, Minji Sohn, Benjamin Pontefract, Michael Klepser

**Affiliations:** Ferris state university college of pharmacy, Lake city, Michigan; Ferris State University, Lake Odessa, Michigan; Ferris State University, Lake Odessa, Michigan; Ferris State University, Lake Odessa, Michigan; Ferris State University, Lake Odessa, Michigan

## Abstract

**Background:**

Introduction: Currently, 25%-40% of all outpatient visits are with physician's assistants (PAs) or nurse practitioners (NPs)1 . PAs and NPs receive less formal education on diagnosis and pharmacology than physicians. The goal of this study was to determine if there is a difference in prescribing patterns between physicians and PAs/NPs for selected infectious diseases.

**Methods:**

Design: This was a cross-sectional study utilizing the CHARM (Collaboration to Harmonize Antimicrobial Registry Measures) database.

**Methods:**

The CHARM database is a Michigan multi-system registry containing archival data previously collected from the medical records of partnering health systems regarding outpatient antibiotic prescribing. This project evaluated antibiotic prescribing data from July 2021 to June 2023 available through CHARM. Antibiotic prescriptions were identified and associated with ICD-10 codes for acute sinusitis, chronic sinusitis, group A streptococcal pharyngitis, UTI, and pyelonephritis. Guideline concordance between prescriber types was compared using chi-square tests.

**Results:**

137,960 prescriptions were included in the study. Of those, 63.7% were written by PAs and NPs, and 36.3% were written by physicians. Compared to physicians, PAs and NPs, prescribed concordant antibiotics more often for acute sinusitis, chronic sinusitis, group A streptococcal pharyngitis, and pyelonephritis (P < 0.01). There was no significant difference between groups for uncomplicated UTIs (P= 0.10).

**Conclusion:**

Our data suggests PAs and NPs prescribe antibiotics at a higher rate of guideline concordance than physicians.

**Disclosures:**

**Minji Sohn, PhD**, Emergent BioSolutions, Inc.: Grant/Research Support

